# Hydrogen cyanide and carboxyhemoglobin assessment in an open space fire‐related fatality

**DOI:** 10.1111/1556-4029.14649

**Published:** 2020-12-28

**Authors:** Daniel Tabian, Diana Bulgaru Iliescu, Tatiana Iov, Barabas Barna, Sebastian Ionut Toma, Gabi Drochioiu

**Affiliations:** ^1^ Faculty of Medicine Transilvania University of Brasov Brasov Romania; ^2^ “Grigore T. Popa” University of Medicine and Pharmacy Iasi Iasi Romania; ^3^ Iasi Institute of Legal Medicine Iasi Romania; ^4^ Faculty of Chemistry “Alexandru Ioan Cuza” University of Iasi Iasi Romania

**Keywords:** carboxyhemoglobin, fire, forensic toxicology, hydrogen cyanide, ninhydrin reagent, postmortem

## Abstract

Hydrogen cyanide (HCN) can be a major contributory factor in death from fire‐related inhalation injury. Although carbon monoxide (CO) is considered the lethal agent of smoke in fires, its liability as a cause of death is sometimes debatable. The purpose of this report is to present the case of an 80‐year‐old man with locomotor disabilities who died due to an open space fire of vegetation debris and household waste in his yard. We evaluated here the concentrations of HCN and carboxyhemoglobin (COHb) and their contribution to the mechanism of death. In addition, the risk factors and the contributing effect of the factors that compose the complex toxic environment that develops in fires were discussed. COHb was determined by spectrophotometry as recommended by Katsumata et al. in 1982. HCN was determined with ninhydrin in postmortem blood samples after removal with 20% phosphoric acid and capture in a potassium carbonate solution. A toxic concentration of 1.3 μg ml^−1^ HCN and a lethal COHb level of 73.7% were determined in the blood samples. Although death was mainly attributed to CO poisoning and extremely severe burns in this open space burning case, the additive effect of HCN in the mechanism of death was also highlighted. The results suggested the possibility that the man's clothing may have played an important role in the production of HCN in this open space fire, as well as other types of garbage that were burned.

**Highlights**

- We found a toxic level of hydrogen cyanide in the blood of a victim of open space fire.
- A lethal concentration of carboxyhemoglobin was measured.
- HCN was determined kinetically spectrophotometrically with ninhydrin.

## 1 INTRODUCTION

Worldwide, fires constitute one of the main sources of public health problems, causing many deaths and injuries [1, 2]. The inhalation of toxic gases that occur during the combustion provokes more fatalities than burns [3, 4]. This is because a fire produces a complex toxic environment composed of flame, heat, lack of oxygen, and smoke with a variety of toxic gases. With an increasing variety of synthetic materials in every house, there is an increasing potential for severe health issues due to the inhalation of smoke [1, 3, 5]. The smoke gas contains carbon monoxide (CO) and hydrogen cyanide (HCN), which are the most significant toxicants but there are more than 400 other toxic constituents known [4, 6, 7]. HCN can be produced by burning natural materials (wool, silk), as well as synthetic materials (polyurethane, polyacrylonitrile, polyamide) [8]. Fire smoke may disable the ability of an individual to escape from the fire, due to the obscuration of vision, irritation, and asphyxia [9]. In terms of how a fire starts, accidents come first in the case of fire‐related disasters, followed by suicide and homicide. Residential home and workplace are the main places where these accidents occur, affecting men and children most of the times [2, 6, 10]. Autopsy reveals characteristic pathological findings like cherry‐red or bright pink livor, bright cherry‐red coloration of blood, musculature, and viscera. A level of COHb higher than 50% in postmortem blood samples is considered to be lethal, as well as a blood concentration of 3 μg ml^−1^ for HCN [11, 12]. Sometimes the responsibility of CO as the cause of death is questionable as the COHb levels are low in postmortem blood samples of fire‐related fatalities [10].

Data in the literature are conflicting as to whether fire deaths are caused by pure HCN intoxication, pure CO intoxication, or mixed HCN‐CO intoxication [13, 14]. There are only a few studies that assessed the HCN in postmortem blood of fire victims, whereas almost no studies are published for open space fire‐related fatalities. Although CO is thought to be the major lethal toxic agent in fires, HCN can be a major contributory factor which causes incapacitation, morbidity, and mortality in domestic fires [15].

It is the aim of this case report to present an open space fire‐related death, where we have assessed the concentrations of HCN and COHb, and their contribution to the mechanism of death. We also discuss the risk factors and the contributive effect of the factors that compose the complex toxic environment which is developed in fires.

## 2 CASE HISTORY

### 2.1 Autopsy findings

We present the case of an 80‐year‐old man with locomotor disabilities, who set fire to garbage in his yard, and his clothes caught fire. Due to the weather conditions, the fire spread. The man managed to move a few meters away from the fire, where he fell in the position in which he was found dead. The autopsy of the deceased man was performed about 24 h after the death. External examination revealed grade III and IV burns on 85%–90% of the body surface, burnt hair follicles on the face, head, and on the rest of the body. Livors were difficult to examine because of the extensive burns; the color was dark bluish red. Internal examination revealed, red hemorrhagic infiltrate of pericranial tissue, free extradural and subdural space, brain edema, and atherosclerotic lesions of the cerebral vessels. The skin of the face showed third degree burns, the pharynx and larynx had a red mucosa, the trachea and bronchi had a violet mucosa, and a dark gray–grayish viscous secretion was observed in the lumen. Additional findings included red color of the blood, lung edema and fatty liver. Atherosclerotic lesions were observed for coronary arteries and large vessels. The esophagus had violet mucosa, and in the lumen, there was moderate partially digested content. About 100 ml of partially digested contents were found in the stomach, while the mucosa was red.

## 3 MATERIALS AND METHOD

### 3.1 Sampling

Toxicological analyses were performed at Toxicology Department of Iasi Institute of Legal Medicine, Romania. Biological samples were collected at autopsy, with ethical committee's approval. CO was assessed by determining the COHb level in 5 ml of femoral venous blood [16]. Ethyl alcohol concentration was evaluated in 5 ml of urine collected at autopsy by puncture of the bladder and 5 ml of femoral venous blood. To determine the concentration of HCN, 5 ml of femoral venous blood was collected in a test tube with sodium fluoride as anticoagulant. The sample was stored at 4°C, and the determination was made 48 h after death.

### 3.2 Methods

All chemical reagents were of analytical grade. Here, we adapted HCN extraction using phosphoric acid solutions [17] and a ninhydrin‐based HCN analysis [18] to blood samples collected in cases of fire. The initial method for determining HCN [18] was slightly modified by replacing sodium carbonate with potassium carbonate to increase the sensitivity of the method. In addition, the absorbance values at 493 nm were measured kinetically spectrophotometrically and not only once at a certain time after mixing the reagents. Thus, in the inner chamber of a Conway cell, 2 ml of 2% K_2_CO_3_ (Merck) was pipetted to capture the HCN released from the outer chamber. In the outer chamber of the cell, 1 ml of blood, 1 ml of MiliQ ultrapure water (Milipore), and 1 ml of 20% H_3_PO_4_ were added. The Conway cell was heated at 40°C for 30 min on an electric heater with automatic shaker system (VMS C1; VWR™). The K_2_CO_3_ solution (the absorbent solution) containing HCN captured after its removal from the 1 ml blood samples was recovered and placed in a 2 ml glass vial. The inner chamber was washed with distilled water to remove the traces of HCN, and the resulting solution was added to the solution found in the glass vial. The resulting volume of absorbent solution was completed with H_2_O to 2 ml and stirred. Then, 100 μl of absorbent was pipetted into the quartz cuvette of the spectrophotometer and treated with 1 ml of ninhydrin reagent (5 mg ml^−1^ ninhydrin in 2% K_2_CO_3_) with stirring. A reference solution was also prepared by mixing 100 μl of 2% K_2_CO_3_ and 1 ml of ninhydrin reagent directly into the spectrophotometric cuvette. The kinetics of the color reaction between cyanide ions and ninhydrin (2,2‐dihydroxy‐1,3‐indandione from Serva) was followed against the reference solution, at 493 nm, using a Libra S35 UV\Visible spectrophotometer provided with 1‐cm quartz cuvettes. Calibration solutions (0; 1.08; 1.625; 2.16 μg ml^−1^ KCN) were prepared by appropriate dilution of the 2.6 μg ml^−1^ KCN stock solution (40 μM KCN, which equates to 1.08 μg ml^−1^ HCN). The calibration solutions were treated similarly with the blood samples to avoid potential errors in the HCN recovery in 2% K_2_CO_3_ solution. Kinetic spectrophotometric measurements were performed for 30 min using Acquire Application Software (Biochrom), and the spectra of real samples were compared with those of the calibration solutions. Unlike the original method in which the absorbance is determined only at 15 min at 485 nm [18], we studied the kinetics of cyanide ion–ninhydrin adduct formation at 493 nm to establish more precisely the value of KCN and, respectively, HCN, knowing that 65 micrograms of KCN equates to 27 μg of HCN. COHb was quantified by UV–vis spectrophotometry [16]. COHb saturation over 50% and HCN level over 3 µg ml^−1^ were considered lethal, while HCN level above 0.5 µg ml^−1^ was considered toxic.

## 4 RESULTS

### 4.1 Chemical analyses

Toxicological examination on blood and urine samples for ethyl alcohol was negative. The COHb level was 73.7%. A blood HCN concentration of 1.3 μg ml^−1^ was measured from the calibration curve carried out with KCN solutions.

**Figure 1:**
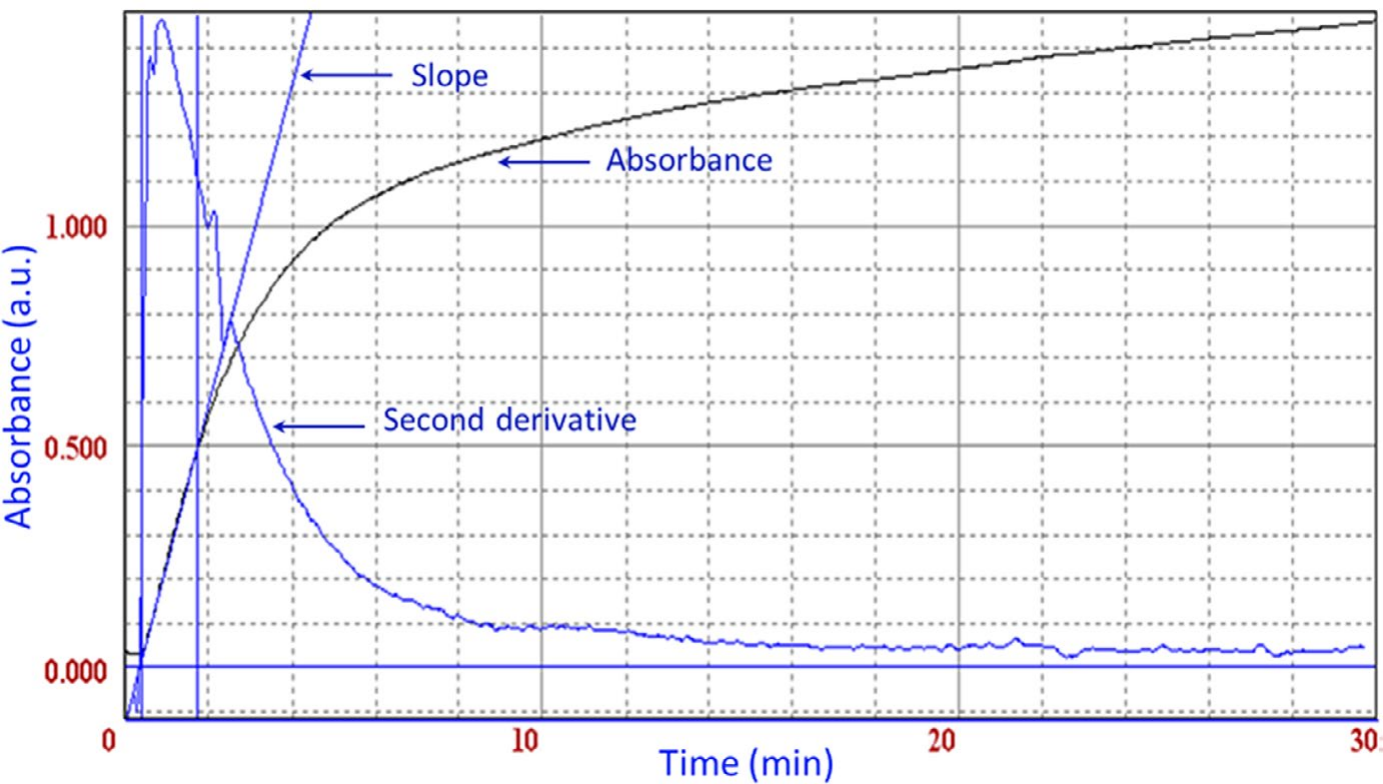
Absorbance values determined at 493 nm and 20°C, which were used to determine the HCN concentration in the blood [Color figure can be viewed at wileyonlinelibrary.com]

The reaction kinetics of HCN absorbed from the blood sample (1 ml) in the K_2_CO_3_ solution (2 ml) was measured spectrophotometrically at 493 nm. Figure 1 shows the kinetics of reaction and the slope as well as the derivative of the absorbance (100 μl of HCN‐containing absorbent and 1 ml of ninhydrin reagent). Maximum absorbance was found at 30 min (A_493nm_ = 1.464). Figure 1 also shows that the absorbance increases greatly in the first 10 min after mixing the two solutions, and then, the growth becomes slowly in the range of 10–30 min. This means that it was mandatory to read the absorbance of a lot of samples over a narrow period of time.

HCN reaction with ninhydrin has been shown to be free of interference when applied to HCN in the blood samples, as the absorbent retains almost all of pure HCN and converts it to KCN. The reaction of HCN in alkaline solutions with ninhydrin is very selective, fast, cheap, being also more sensitive than most available colorimetric methods [18].

A close correlation was found between the absorbance values determined with the kinetic spectrophotometric method and those measured with the old analytical procedure (Figure S1). When comparing the absorbance values of the HCN–ninhydrin adduct, under the conditions of the kinetic spectrophotometric method, with those obtained with the original method, the closest values were found at minute 30 (Table S1). From the calibration curve (Figure S2), a blood HCN concentration of 1.3 μg ml^−1^ was obtained. Moreover, with the help of this curve one can find directly the HCN concentration in the absorbent solution, or the regression equation *y* = 0.3956*x* + 0.0595 can also be used, where *y* is the HCN concentration, and *x*, the absorbance at 493 nm, measured after 30 min on the kinetic curve (*r* = 0.984***; *R* ^2^ = 0.9688). The HCN concentration of the absorbent solution should multiplied by 2 to obtain the HCN concentration in the blood because HCN from 1 ml of blood was captured in 2 ml of K_2_CO_3_ solution.

## 5 DISCUSSION

For each fire death, an autopsy is performed and the first question that arises is whether the victim was exposed to the fire while he was alive and breathing. If the victim was exposed to the fire after death, we may find that the fire was used to camouflage a prior death. Presence of CO in blood measured as COHb concentration above 10% is a biomarker of smoke inhalation [2, 3]. In the reported case, a concentration above 73% was measured, suggesting a lethal dose. However, if HCN is present at toxic concentrations, it can indirectly produce injury or death, by causing confusion and incapacitation to act, that delay or prevent escape from a fire, and prolongs time of exposure [4, 19]. Geldner reported that 60% of patients were able to escape from the fire scene, while 39% of them had to be rescued. HCN level was significantly higher in patients who had to be rescued. In the same study, a correlation between the Glasgow Coma Scale score (GCS) and HCN concentration was observed. A correlation between the GCS score and the blood COHb concentrations was also reported [3].

Usually, HCN intoxication occurs in enclosed‐space fires [4]. It is known that fire smoke produced in combustion of wood does not contain HCN. The case we talk about is atypical and raises a lot of questions because combustion of vegetation debris, victim's clothes and of household waste in open space liberated HCN at high concentration, which was found in the blood of the deceased. Frequently, in rural areas from Romania, people burn plastic bottles, and other house waste made from plastics, along with vegetation debris, resulting in high amounts of HCN liberated in the fire smoke. Materials such as wool, silk, polyurethane, polyamide, and polyacrylonitrile are known to produce HCN when combusted [8]. The victim's clothing and body were extensively burned, and the extremely high COHb saturation indicates that much of the combustion of the clothing likely occurred while the man was alive. Taking these into account, we can suggest that the man's clothing could have played an important role in the production of HCN in the reported open space fire.

The lethal COHb concentrations frequently lead to an inability to confirm the presence of other toxicants, because of the perception of CO as the most significant contributor to smoke inhalation‐associated morbidity and mortality [19]. However, high amounts of HCN are reported to be present in the blood of fire survivors and fatalities [20]. Eckstein claims that HCN toxic‐to‐lethal levels are found in approximately 33% to 90% of fire‐related fatalities in closed‐space fires. Moreover, HCN has proven to be the primary cause of death from smoke inhalation in some cases [19].

In open space fires, smoke can be a problem to individuals who are more sensitive. Meteorological conditions and extreme weather events like wind are further increasing the risk to health, as fire extends or increases in intensity [1]. The elderly are at a higher risk because of their reduced ability to escape, considering old age and/or possible locomotor disabilities. In our case, the victim had compromised mobility issues that would make him less capable of escape from a fire that progressed faster than he anticipated. The COHb saturation indicates that he survived long enough to inhale a substantial amount of smoke. Another group at higher risk for fire‐related morbidity and mortality includes people with preexisting cardiovascular and respiratory diseases [4]. In a study published by Stoll, almost half of the individuals included were over 70 years old, and they had preexisting cardiac conditions such as stenosis of the coronary arteries and myocardium hypertrophy and/or callosities. Stoll reported lower concentrations for COHb and HCN in the group of people over 70 years old who died, which can be explained by contribution of heart and pulmonary conditions in the mechanism of death [4]. In our report, internal examination revealed also atherosclerotic lesions in the coronary arteries and large vessels.

CO can be assessed in most hospitals and legal medicine services because the concentration of COHb can be measured quickly and easily using blood gas analysis [16]. Although some combustible materials release a considerable amount of HCN, diagnosis of poisoning is hampered by a lack of awareness that smoke caused by fires is an important source of HCN. HCN blood concentration is not a routine investigation because of the absence of a rapidly returnable diagnostic test, and in most hospitals, medical laboratories, and toxicology departments, it is not performed [4, 12, 19]. In hospitals and legal medicine services from Romania, the determination of HCN is not included in the routine toxicological analysis for fire victims, as COHb level and alcohol concentration are.

Thermal lesions found at autopsy guided us to the specific request for toxicological analysis. CO was suspected, with direct action or together with other factors such as burnings and lack of oxygen [2]. Burnings and traumatic factors are also incriminated in literature [2]. In our case, the autopsy revealed 3rd and 4th degree burns on 85%–90% body surface. Death was attributed to the extensive severe burns, and COHb level has been interpreted as a vital sign.

The degree to which HCN contributes to smoke inhalation‐associated morbidity and mortality has proven difficult to measure. An important factor for HCN analysis is the time elapsed from death to autopsy and blood sampling. Another factor that influences the postmortem variation of HCN is the storage temperature, time between blood sampling and assay, methemoglobin content of sampled blood and COHb saturation of sampled blood. However, there is still little‐known information about the disappearance of HCN from blood and it is difficult to estimate the blood HCN level at the time of death from measurements made many hours or even days later. Assessment of CO in the blood is not associated with these difficulties. One shortcoming in the present study is that the time elapsed from sampling to measurements is about 24 h. Nevertheless, we found toxic concentration of HCN in the postmortem blood sample.

In a study on the cardiac arrest of 5 people exposed to smoke from closed fire, concentrations higher than 3 µg ml^−1^ HCN were found in 3 cases, 2 of them having COHb concentration under 50%. The authors discussed that cardiac arrest was more likely to have been provoked by HCN intoxication rather than CO [12]. Stoll showed that in only 3 of the 45 cases HCN could not be detected [4]. In that study, HCN level was measured for 92 blood samples of fire or smoke gases‐related fatalities, and the highest concentrations of HCN were found after enclosed‐space fires. A lethal concentration was determined in 6 cases. The authors also reported 3 cases in which HCN was at lethal concentration, while COHb levels were under 50% and they concluded that CN was the cause of death in these cases. In the same study, they report a mixed‐lethal intoxication, as the HCN and COHb levels alone cannot explain the lethal result in 12 cases. In only 3 of the 45 cases, HCN could not be detected [4]. On this line, Geldner reported a significant correlation between blood HCN and COHb concentration. Stamyr also discussed a possible additive effect of HCN and CO, suggesting that death is in most cases not caused by CO alone. Gill et al. conducted a study on 87 fire‐related fatalities at a club in New York City, and he reported that 97% of the victims had a COHb level over 50%, whereas HCN blood concentrations ranged from undetected to 5.5 µg ml^−1^ [21, 22].

## 6 CONCLUSIONS

We introduced a case of open space fire‐related fatality in which we measured a toxic level of HCN and a lethal concentration of COHb, as well as other factors commonly encountered in fires, such as burns and lack of oxygen. This raises a warning whether HCN should be regularly assessed for fire victims, coming from open space fires but more specifically, coming from enclosed‐space fires. Elderly, people with locomotor disabilities or other associated conditions, as preexisting cardiovascular or pulmonary diseases, are at a higher risk for fire‐related morbidity and mortality. HCN poisoning can be detected in postmortem blood samples using the quantitative method described in this report.

## Supporting information

Supplementary Material
