# Supplementary material for: Hydrogen cyanide and carboxyhemoglobin assessment in an open space fire‐related fatality
Source: J Forensic Sci. 2020 Dec 28;66(3):1171–5. doi: 10.1111/1556-4029.14649 (PMC8246848; doi:10.1111/1556-4029.14649)
Supplement: Supplementary file 1 — Supplementary Material [file JFO-66-1171-s001.docx]

**SUPPLEMENTAL INFORMATION**

**Hydrogen cyanide and carboxyhemoglobin assessment in an open space fire-related fatality**

Tabian D, Bulgaru Iliescu D, Iov T, Barna B, Toma SI, Drochioiu G

A kinetic-spectrophotometric test based on the reaction of ninhydrin with HCN was applied to determine HCN in post-mortem blood samples after removal with 20% phosphoric acid and capture in a 2% solution of K_2_CO_3_.

Both Figure S1 and Table S1 show that the improved version of the method for determining HCN with ninhydrin produces results similar to the original. The regression equation was y = 0.9128·x, where y is the absorbance at 485 nm, and x the absorbance at 493 nm, taken at 30 min from the kinetics spectrum (Figure S1). A highly significant correlation coefficient, r = 0.995^***^ was calculated (R² = 0.9904), which suggests that the results obtained with the two methods are similar.

FIGURE S1 *Close correlation between the absorbance values determined with the method with sodium carbonate at 485 nm (at 15 min) and those measured using the kinetic spectrophotometric method at 493 nm, at 30 min.*

FIGURE S2 *Calibration curves for cyanide determination (absorbance values at 30 min): (a) absorbance versus concentration on using the absorbent solutions only; (b) calibration curve for calculation of HCN concentrations in blood samples*.

In addition, we compared the values for the absorbance of the HCN-ninhydrin adduct measured at 493 nm (at 10 min, 20 min and 30 min, respectively), in the conditions of the kinetic spectrophotometric method, with those obtained with the original method, based on a single spectrophotometric reading at 485 nm, and the use of sodium carbonate instead of K_2_CO_3_ (Table S1). As expected, the absorbance at 485 nm measured 15 min after the addition of the reagents was slightly lower than that of the kinetic method, which was measured at 493 nm.

TABLE S1 *Comparative measurements of HCN-ninhydrin adduct absorbance at 493 nm (at 10 min, 20 min and 30 min, respectively, according to the kinetic spectrophotometric method) with that of the old method (a single spectrophotometric reading at 485 nm, and the use of sodium carbonate instead of potassium carbonate)*.

| Concentration of  KCN (μg mL^-1^) | Absorbance  (493 nm; 10 min) | Absorbance  (493 nm; 20 min) | Absorbance  (493 nm; 30 min) | Absorbance  (485 nm; 15 min) |
| --- | --- | --- | --- | --- |
| 0.000 | 0.000 | 0.000 | 0.000 | 0.000 |
| 0.325 | 0.205 | 0.160 | 0.160 | 0.170 |
| 0.650 | 0.680 | 0.690 | 0.590 | 0.630 |
| 0.650 | 0.730 | 0.740 | 0.790 | 0.675 |
| 0.975 | 1.025 | 1.070 | 1.100 | 0.965 |
| 1.300 | 1.285 | 1.320 | 1.290 | 1.190 |
| 1.300 | 1.295 | 1.300 | 1.310 | 1.200 |

Due to the high sensitivity of the HCN reaction with ninhydrin, the absorbance exceeded the unit value by less than 1 μg mL^-1^ of HCN. We expressed HCN concentrations as either μg mL^-1^ of HCN or μg mL^-1^ of KCN, depending on the experiment performed. Its remarkable sensitivity was also indicated by measuring very small volumes (100 μL) of absorbent or artificial solution.

The following regression equations and correlation coefficients were calculated: y = 0.8086x + 0.1444 & r = 0.965, y = 0.9079x + 0.0714 & r = 0.990, y = 0.982x + 0.0242 & r = 0.991, where y was the absorbance values at 493 nm and at min 10, min 20, and min 30, respectively. The absorbance at 485 nm measured in the presence of sodium carbonate was denoted by x. The kinetic spectrophotometric method provides similar results to the original.
